# Building the political case for investing in public health and public health research

**DOI:** 10.17269/s41997-019-00214-3

**Published:** 2019-05-06

**Authors:** Steven J. Hoffman, Maria I. Creatore, Ariane Klassen, A. Morgan Lay, Patrick Fafard

**Affiliations:** 1 CIHR Institute of Population & Public Health, Canadian Institutes of Health Research, 2120 Dahdaleh Building, 4700 Keele Street, Toronto, ON M3J 1P3 Canada; 2 Global Strategy Lab, York University/University of Ottawa, Ottawa, Canada; 3 Dahdaleh Institute for Global Health Research, Faculty of Health and Osgoode Hall Law School, York University, Toronto, ON Canada; 4 Department of Health Research Methods, Evidence & Impact and McMaster Health Forum, McMaster University, Hamilton, ON Canada; 5 Department of Global Health & Population, Harvard T.H. Chan School of Public Health, Harvard University, Boston, MA USA; 6 Division of Epidemiology, Dalla Lana School of Public Health, University of Toronto, Toronto, ON Canada; 7 Graduate School of Public & International Affairs, Faculty of Social Science, University of Ottawa, Ottawa, ON Canada; 8 Department of Science, Technology, Engineering & Public Policy, University College London, London, UK

**Keywords:** Public health, Evidence-based policy, Knowledge translation, Policy making, Politics, Santé publique, Politiques factuelles, Application des connaissances, Élaboration des politiques, Politique

## Abstract

Governments around the world vastly underinvest in public health, despite ever growing evidence demonstrating its economic and social benefits. Challenges in securing greater public health investment largely stem from the necessity for governments to demonstrate visible impacts within an election cycle, whereas public health initiatives operate over the long term and generally involve prevention, statistical lives and underlying conditions. It is time for the public health community to rethink its strategies and craft political wins by building a political case for investing in public health—which extends far beyond mere economic and social arguments. These strategies need to make public health visible, account for the complexities of policymaking networks and adapt knowledge translation efforts to the appropriate policy instrument.

## Résumé

Partout dans le monde, malgré une démonstration de mieux en mieux étayée des avantages économiques et sociaux que procurent les investissements en santé publique, les gouvernements négligent trop souvent ce secteur. Les difficultés à obtenir des investissements accrus tiennent en grande partie à la nécessité pour les gouvernements de démontrer des effets visibles à l’intérieur d’un cycle électoral, alors que les initiatives de santé publique fonctionnent sur le long terme et sont généralement affaire de prévention, de vies statistiques et de conditions sous-jacentes. Le temps est venu pour le milieu de la santé publique de repenser ses stratégies et de créer des victoires politiques en élaborant une argumentation en faveur de l’investissement en santé publique – qui va bien au-delà des simples considérations économiques et sociales. Ces stratégies doivent rendre la santé publique visible, tenir compte de la complexité des réseaux d’élaboration des politiques et adapter les efforts d’application des connaissances à l’instrument politique approprié.

## The world massively underinvests in public health

Governments around the world underinvest in public health and public health research. Across member countries of the Organisation for Economic Co-operation and Development (OECD), the share of health expenditures for prevention rarely exceeds 6% (Gmeinder et al. 2017). In Canada, a mere 5.5% of total health spending is on public health. Included in this estimate is a variety of services, including food and drug safety, health inspection, health promotion, occupational health, community mental healthcare, and services delivered by public health nurses (Canadian Institute for Health Information 2017). Public health research fares only slightly better, attracting 9% of funding from the Canadian Institutes of Health Research (Canadian Institutes of Health Research 2018).

Despite growing investment in healthcare, we are starting to see the limits of our physician- and hospital-centred health system. Although we are living longer than ever before, the proportion of the population living with chronic disease, disability and mental health challenges is increasing in Canada. The 2017 Canadian Chronic Disease Indicators highlight that 28.1% of adults aged 18 years or older are considered obese and that nearly one in eight (12.2%) Canadians over the age of 12 have been diagnosed with mood or anxiety disorders (CCDI Steering Committee 2017). More concerning still is that the increases in obesity, anxiety and depression are also seen among children and youth, changing the societal burden of chronic disease from one largely experienced by the elderly to one experienced across all demographics (Tremblay et al. 2010).

Our collective health depends on our ability to convince decision-makers that investing in public health is a politically smart choice. While the public health community has repeatedly demonstrated the economic and social value of public health approaches (Masters et al. 2017), government budgets for prevention and public health have not significantly increased. Many have even argued that public health in Canada is under attack—both in the form of budget cuts and institutional restructuring (Potvin 2014; Hancock 2017). So, while public health is well positioned to respond to today’s health challenges, we need to work together to make a clearer case for investment and secure the necessary resources for a healthier Canada.

## Four reasons why public health is tough politics

The prolonged petrification of public health budgets is, paradoxically, prudent politics. There are numerous reasons why economic and social arguments alone have failed to motivate political action.

Most important to consider is that, with some notable exceptions like pensions and the environment (Jacobs 2011), politicians in democratic societies typically operate within the constraints of the election cycle. Priorities and investments are necessarily shaped by a need to garner votes, gain recognition and establish a legacy. In order to maintain and build electoral support, politicians need projects and policies that are visible and have demonstrable effects within their time in office. Democratic politics can create political realities that favour short-term “quick wins” over long-term sustained impact.

Within this system, there are at least four reasons why investing in public health is not an obvious political win.

First, *public health typically focuses on prevention rather than treatment*. The bulk of our health spending goes towards treating illness faced by real people today (a tangible outcome) rather than preventing illness for people in the future (an abstract outcome). The prevention agenda is difficult to sell politically because we may not personally see results or may not recognize public health at work. For example, someone who has not experienced tooth decay is unlikely to attribute this positive dental health outcome to fluoridation in their drinking water.

Second, *public health typically improves statistical lives rather than individual lives*. Whereas patients are living, breathing, success stories who can testify to the importance of treatment, the beneficiaries of population-level interventions are not always identifiable. Without faces, names and testimonials, it is difficult to communicate the benefits of public health to voters.

Third, *public health successes are typically achieved over the long term rather than within short election cycles*. Governments may be reluctant to bear the immediate costs of public health when the benefits and recognition will be reaped by the next elected government.

Fourth, by focusing on prevention, *public health typically addresses the underlying conditions that lead to ill health*. Strategies used in public health often encourage system, environment and behaviour change. Creating the conditions for good health is complex and multisectoral. Drawing the link between public health strategies at a societal level and improved outcomes is methodologically and rhetorically more difficult.

## Public health faces funding crises except during crises

These obstacles make it more difficult to get and keep public health at the top of the political agenda. Only in times of crisis does public health emerge as a priority, and often the attention is fleeting.

Take the World Health Organization’s budget for disease outbreak response as an example (Hoffman and Røttingen 2014; Hoffman 2010). Substantial increases to emergency funds followed the 2003 SARS outbreak, the 2006–2007 peak of the H5N1 human cases, the 2009 H1N1 pandemic and the 2014 Ebola outbreak. But only 2 years after SARS, the emergency response budget decreased by 5%; 3 years after H1N1, it was cut by 25% (World Health Organization 2013, 2005). Funding for public health is all too often reactionary and unreliable.

It is difficult to know exactly how to overcome the political obstacles that public health faces. At the very least, a change in tactics is probably necessary. Together, we can work to craft political wins by shifting the focus beyond the narrow economic case towards a broader political investment case. Economic arguments remain necessary, but increasing visibility and developing an understanding of democratic politics are equally important to demonstrating the value of public health.

## It is time to hone the political investment case for public health

If we are to craft a convincing political case, the public health community must build on existing knowledge and expertise to develop a more sophisticated understanding of how the political system works (Greer et al. 2017; Hoffman and Silverberg 2015). Three strategies can help make public health a political win for today’s politicians. We need to (1) make public health visible, (2) navigate diffused and non-linear decision-making processes and (3) differentiate between policy instruments (Fafard and Hoffman 2018).

1. Make public health visible

We can help make the political case for public health by highlighting our impact on citizens’ day-to-day lives. From routine activities, including immunization and food safety inspection, to activities that extend beyond what the public often thinks of as public health—including safe workplace regulations, pollution controls, traffic speed limits and gun restrictions—we need to consistently remind Canadians that the impacts of public health are everywhere. It is time for public health to celebrate its achievements, brand its efforts and build awareness for its contributions. Public health researchers and practitioners should consistently bring their evidence and expertise to bear on public debates and act to correct misinformation. Speaking publicly about our collective accomplishments and value will strengthen our collective political case for investment.

2) Navigate diffused and non-linear decision-making processes

The next step is to demonstrate value to politicians while accounting for the complex political realities that they face. Though many public health practitioners engage in the politics of public health on a daily basis, the public health community as a whole—researchers, practitioners and stakeholders—needs to develop a more sophisticated appreciation of the limits of scientific evidence as well as who policymakers are and how they are organized. For each public health issue, we need to consider that policy actors are diverse, usually diffuse, and often embedded in hierarchical policy networks (Fafard and Hoffman 2018).

More specifically, efforts to demonstrate the value of public health and influence decision-making should be tailored to the characteristics of the policy network and advisory system that exist within and across governments (Hoffman et al. 2018a, b; Groux et al. 2018; Behdinan et al. 2018). For example, the type and composition of the policy network updating school-based immunization policies is likely much smaller and less diverse in opinion and approach than the network of actors working to reduce childhood obesity (Hoffman and Silverberg 2015). These networks also differ in the types of non-governmental and non-health actors involved. Mobilization on childhood obesity will naturally overlap with education, social services, food regulation and taxation, and will involve a diverse set of civil society actors mobilized around the issue. To demonstrate its value, public health needs to increase engagement with this complexity and learn to navigate this system of diffused and non-linear decision-making.

3) Differentiate between policy instruments

Finally, when conducting research and designing solutions, public health researchers, practitioners and stakeholders need to consider more critically the type of policy instrument that is most appropriate for the issue they are addressing. Policymakers depend largely on four ways of achieving their policy objectives: (1) regulation, (2) communication, (3) taxation and (4) spending (see Table 1) (Fafard and Hoffman 2018). Each policy instrument requires a tailored strategy that considers the differing processes for policy development and implementation and the corresponding opportunities for the public health community to intervene. For example, public health initiatives that include new or amended legislation or regulation often include formal consultation processes, while public awareness or spending initiatives might be conceived of and designed with little opportunity for formal feedback. These more internal and opaque decision-making processes require public health researchers, practitioners and stakeholders to build relationships with policy actors and strategize with partners to communicate a clear message to multiple audiences.

**Table 1:** Implications of different policy instruments for KT approaches (Source: Fafard P, Hoffman SJ. Rethinking knowledge translation for public health policy. *Evidence & Policy* 2018; 1–11. doi:10.1332/174426418X15212871808802)

Policy instrument	Implications for KT approaches
a) Regulating (for example, food safety regulations)	• Regulators often rely on highly structured processes of consultation and input such as regulatory hearings, which may supersede dyadic KT approaches.
	• Decisions might be subject to judicial review such that regulatory processes often discourage informal or non-transparent inputs.
b) Communicating (for example, healthy food guides)	• Communications can come from different kinds of actors and in different forms, from elected politicians giving speeches to public servants publishing documents.
	• Traditionally, public health officials have wide decisional authority on the messages they communicate, especially on highly technical matters and during crises.
	• A wider range of economic departments and central policy agencies will be involved when messages could create economic winners and losers.
c) Taxing (for example, tobacco taxes)	• Ministries of finance and central agencies usually control taxation. • Tax policy creates winners and losers, attracts significant lobbying from powerful stakeholders, and is rife with follow-on consequences.
	• Proposed public health policies are often contested on ideological grounds and actors who stand to be taxed may seek to undermine public health research.
d) Spending (for example, free vaccinations)	• Ministries of finance and central agencies ultimately control budgeting, although budget requests typically originate in line departments.
	• Public health officials’ control over spending decisions typically increases as those decisions move from broad policy objectives towards specific implementation of those policies; in turn, scientific evidence may become even more influential.
	• Some measures that would improve population health (for example, reducing income inequality by means of redistribution) are very costly and, absent tax increases, mean less money for other priorities.

## Conclusion

Public health and public health research are pivotal for tackling many of the most pressing issues facing society today. Because the work we do extends beyond any given election cycle, public health faces challenges to becoming and staying a political priority. By making public health visible, navigating the complex political realities of policymaking and differentiating between policy instruments, we can collectively hone the political case for public health investment. Canadians and people around the world are depending on us to do so.
